# Consumption

**Published:** 1886-06

**Authors:** 


### CONSUMPTION.

Dr. McCoskey, Professor of diseases of the throat and chest, Fort Wayne (Ind.) College of Medicines, contributes a very excellent article to the *Fort Wayne Journal of Medical Sciences*, on the Geographical Pathology of Consumption. He says that in 1880, the deaths from this disease in the United States numbered 90,551 nearly one-eighth of the mortality from all other causes. Making due allowance for our defective system of registration and the still greater defect in some of our physicians who pronounce many diseases which they cannot cure and with which they have formed no acquaintance, consumption, the tables are valuable to all and they are perfect enough to show very plainly where the disease is most prevalent.

With the exception of the States California, Florida, Louisana and Pennsylvania, and the Territories, Arizona, Nevada and Montana, the death-rate from all causes was larger in 1880 than in 1870 The result of this increase upon the part of the individual State is to raise the death-rate of the United States from 13.3 per 1,000 in 1870 to 15.1 in 1880, an increase of 13½ per cent. Curiously contrasting with this fact, the death-rate from consumption either shows no change or a slight increase in all except fifteen States and Territories. The average for the United States, which shows no change whatever, remaining 1.8 per 1,000 for each year, only illustrates and confirms the remark that local variations neutralize one another, thus making no change in the general result. The following table has been compiled from the census report of 1860 and 1870 :

*Showing Death-rate* (1) *from all Causes,* (2) *from Consumption, and* (3) *Percentage of Latter to Former.*

| | Deaths per 1,000 of Population. | | Deaths from Consumption per 1,000 of Population. | | Percentage of Deaths from Consumption to all Deaths. | |
|---|---|---|---|---|---|---|
| | 1870 | 1880 | 1870 | 1880 | 1870 | 1880 |
| UNITED STATES | 13.3 | 15.1 | 1.8 | 1.8 | 14.1 | 12.1 |
| Alabama | 10.8 | 14.2 | ·76 | 1.4 | 7.0 | 9.0 |
| Arizona | 26.1 | 7.2 | 0.1 | .4 | .4 | 6.9 |
| Arkansas | 12.6 | 18.4 | .9 | 1.2 | 7.0 | 6.4 |
| California | 16.1 | 13.4 | 2 2 | 2.2 | 13.8 | 15.6 |
| Colorado | 9.4 | 13.1 | 0.8 | 1.1 | 8.5 | 8.2 |
| Connecticut | 12.6 | 14.7 | 2.3 | 2.2 | 17.9 | 15.2 |
| Dakota | 7.1 | 9.6 | .9 | .8 | 12.8 | 8.9 |
| Delaware | 12.5 | 15.1 | 2.4 | 2.4 | 18.9 | 11.1 |
| District of Columbia | 15.3 | 24.5 | 3.4 | 4.4 | 21.9 | 19.0 |
| Florida | 12.1 | 11.7 | 0.7 | 1.0 | 5.7 | 8 0 |
| Georgia | 11.5 | 13 9 | 0.7 | 1.1 | 6.4 | 8.0 |
| Idaho | 3.3 | 9.1 | 0 3 | 0.7 | 10.0 | 6.1 |
| Illinois | 13.3 | 14 5 | 1.4 | 1.5 | 10.8 | 13.3 |
| Indiana | 10.5 | 15.7 | 1.7 | 2.0 | 15.5 | 12.7 |
| Iowa | 8.1 | 11.9 | 1.1 | 1.2 | 13.7 | 9 9 |
| Kansas | 12.5 | 15.2 | 1.1 | 1.1 | 9.0 | 7.3 |
| Kentucky | 10.9 | 14.4 | 1.9 | 2.3 | 17.4 | 15.7 |
| Louisiana | 20.0 | 15.4 | 1.9 | 1.6 | 9.7 | 10.7 |
| Maine | 13.3 | 14.8 | 3.2 | 2.8 | 25 6 | 19.2 |
| Maryland | 12.4 | 18.1 | 2.1 | 2.5 | 17.2 | 14.1 |
| Massachusetts | 17 7 | 18.5 | 3.5 | 2.9 | 19.9 | 15.7 |
| Michigan | 9.4 | 12.0 | 1.6 | 1.6 | 16.4 | 13.2 |
| Minnesota | 8.0 | 11.6 | 1.0 | 1.1 | 13.0 | 9.4 |
| Mississippi | 11.1 | 12.9 | .8 | 1.1 | 7.6 | 8.2 |
| Missouri | 16.3 | 16.9 | 1.6 | 1.7 | 9.7 | 9.1 |
| Montana | 9.0 | 8.0 | .8 | .5 | 9.2 | 5.4 |
| Nebraska | 8.1 | 13.1 | .7 | .9 | 8 7 | 7.0 |
| Nevada | 14.5 | 17.7 | .7 | 1.0 | 4.8 | 8.4 |
| New Hampshire | 13.5 | 16.1 | 3.0 | 2.5 | 22.2 | 15.7 |
| New Jersey | 11.7 | 16.3 | 2.0 | 2.3 | 17.2 | 14.2 |
| New Mexico | 12.8 | 20.3 | .5 | .4 | 3.8 | 2.5 |
| New York | 15.8 | 17.4 | 2.6 | 2.5 | 16.7 | 14.6 |
| North Carolina | 9.8 | 15.4 | 1.1 | 1.5 | 11 7 | 9.1 |
| Ohio | 11.1 | 13.3 | 2.0 | 1.9 | 17.7 | 13.9 |
| Oregon | 6.9 | 10.7 | 1.2 | 1.3 | 18.0 | 12.1 |
| Pennsylvania | 14.9 | 10.2 | 2.1 | 1.9 | 14.2 | 15.6 |
| Rhode Island | 12.6 | 17.0 | 2.5 | 2.4 | 20.1 | 14.7 |
| South Carolina | 10.5 | 15.8 | .9 | 1.6 | 8.9 | 9.1 |
| Tennessee | 11.3 | 16.8 | 1.9 | 2.4 | 16.7 | 14.5 |
| Texas | 13.7 | 15 5 | .8 | 1.0 | 5.9 | 6.6 |
| Utah | 10.3 | 16.7 | .7 | .5 | 7 1 | 2.9 |
| Vermont | 10.7 | 15.1 | 2.2 | 2.4 | 20.2 | 16 0 |
| Virginia | 12.4 | 16 3 | 1.7 | 2.0 | 13.8 | 12.1 |
| Washington | 9.3 | 10.5 | 1.5 | 1.3 | 15.7 | 13.2 |
| West Virginia | 9 1 | 11.9 | 1.6 | 1.6 | 17.6 | 13.1 |
| Wisconsin | 9.4 | 12.1 | 1.2 | 1.3 | 13.2 | 10.5 |
| Wyoming | 8.1 | 9.1 | .4 | .3 | 5.4 | 2.6 |

The well-known prevalence of the disease in New England, and its rarity in the South Atlantic and Gulf coast States, become at once apparant. It is also observed that, with numerous undulations, it is somewhat evenly distributed over the remaining States east of the 95th meridian. In the vast region west of this meridian, comprising more than one-half the territory of the United States, and about one-fifteenth of its popula ion, it is, with the exception of a portion of California (Texas is included in another division) even less prevalent than in the South Atlantic and Gulf coast States." * * * *

On the contrary, when we compare the Interior and lake States as far west as Kansas and Nebraska in different latitudes under the same meridian, we find a general increase in the consumption death-rate as we pass from North to South. Taking the States traversed by the 85th meridian, the death-rate per one thousand of the population for 1880, were as follows: Michigan, 1.6; Indiana, 2.0; (Ohio by its side, 1.9); Kentucky, 2.3; and Tennessee, 2.5. In 1870, however, no difference was reported between Kentucky and Tennessee. Again, taking the States traversed by the 85th meridian, the death-rates are as follows: Minnesota, 1.1; Iowa, 1.2; Missouri, 1.7; but here the rise ceases, and Arkansas falls to 1.2, while its mean annual temperature is about 20 degrees Fahr. higher than that of Minnesota. On the 100th meridian it is as follows: Dakota, .8; Nebraska, .9; and Kansas, 1.1, thus showing a slight, gradual increase with the rise of temperature. But the relative positions of Dakota and Nebraska in the scale, were reversed in 1870; while Arizona and New Mexico, with about the same temperature as Tenessee, have about the lowest death rate in the United States; while Tenessee has nearly the highest. Southern California, with a mean annual temperature considerably higher than the northern portion of the State, is almost exempt from the disease, while it is very prevalent in the latter portion of the State. Oregon and Washington Territory, with a temperature more than 20 degrees lower than that of Arizona, have a mortality from consumption more than three times as great.

What, then, are the inferences to be drawn from these facts, regarding the etiological relation of different mean annual temperatures to consumption as indicated by its mortality? From the Atlantic coast alone we are justified in concluding that consumption diminishes almost *pari passu*, with a rise of temperature. This is rather the statement of a fact than an inference. But, on the other hand, that temperature alone is either unimportant or entirely masked by other conditions, is abundantly shown. The same isothermal belt, for instance, traverses Maine and Minnesota; the consumption death-rate of the latter being about one-third that of the former. That the difference is not dependent upon the general healthfulness of Minnesota is conclusively proven by the fact that its death-rate from all causes is three-fourths that of Maine. The question of temperature is almost inextricably complicated with other atmospheric conditions of scarcely less importance.

We have not room for the whole of this interesting article, but it shows most conclusively that there is no climate wholly free from consumption, and it shows another fact, that our physicians have yet much to study and learn in regard to that disease. It concludes as follows:

"The amount of moisture in the air was estimated comparatively by the annual rain-fall. This standard, while not always correct, was considered sufficiently so for purposes of comparison.

It was found that there was no constant relation between certain degrees of atmospheric moisture and the mortality from consumption. The two regions where the mortality is less than in any other part of the United States, (Gulf States and Colorado regions) have respectively the highest and lowest rainfall, being as high as eighty inches in the former and as low as eight in the latter.

The effect of moisture appears very different as it is associated with a high or low temperature. With a high temperature it is rather favorable than otherwise, dryness promoting irritation of respiratory mucous membrane (Jaccoud.) With low temperature it is prejudicial, as illustrated in New England.

As all grades of mortality from consumption were found on the sea coast, from the infected regions of New England to the health resorts of Florida and Southern California, it was concluded that there could not be, as stated by Professor Flint, a uniformly greater prevalence of consumption on the sea coast."

